# Supplementary material for: Synthesis of Poly(Malic Acid) Derivatives End-Functionalized with Peptides and Preparation of Biocompatible Nanoparticles to Target Hepatoma Cells
Source: Nanomaterials (Basel). 2021 Apr 9;11(4):958. doi: 10.3390/nano11040958 (PMC8070460; doi:10.3390/nano11040958)
Supplement: Supplementary file 1 [file nanomaterials-11-00958-s001.pdf]

Supplementary files

Synthesis of Poly(Malic Acid) Derivatives End-Functionalized with Peptides and Preparation of Biocompatible Nanoparticles to Target Hepatoma Cells

Clarisse Brossard [1], Manuel Vlach [2], Elise Vène [2,3], Catherine Ribault [2], Vincent Dorcet [1], Nicolas Noiret [1], Pascal Loyer [2,*], Nicolas Lepareur [2,4,*] and Sandrine Cammas-Marion [1,2,*]

[1] Univ Rennes, Ecole Nationale Supérieure de Chimie de Rennes, CNRS, ISCR, UMR 6226, ScanMAT, UMS2001, F-35000 Rennes, France; clarisse.brossard@ensc-rennes.fr (C.B.); vincent.dorcet@univ-rennes1.fr (V.D.); nicolas.noiret@ensc-rennes.fr (N.N.)

[2] INSERM, INRAE, University Rennes, Institut NUMECAN (Nutrition Metabolisms and Cancer) UMR_A 1341, UMR_S 1241, F-35000 Rennes, France; manuel.vlach@univ-rennes1.fr (M.V.); elise.vene@univ-rennes1.fr (E.V.); catherine.ribault@univ-rennes1.fr (C.R.)

[3] Pôle Pharmacie, Service Hospitalo-Universitaire de Pharmacie, CHU Rennes, F-35033 Rennes, France

[4] Comprehensive Cancer Center Eugène Marquis, F-35000 Rennes, France

* Correspondence: pascal.loyer@univ-rennes1.fr (P.L.); n.lepareur@rennes.unicancer.fr (N.L.); sandrine.marion.1@ensc-rennes.fr (S.C.-M.)

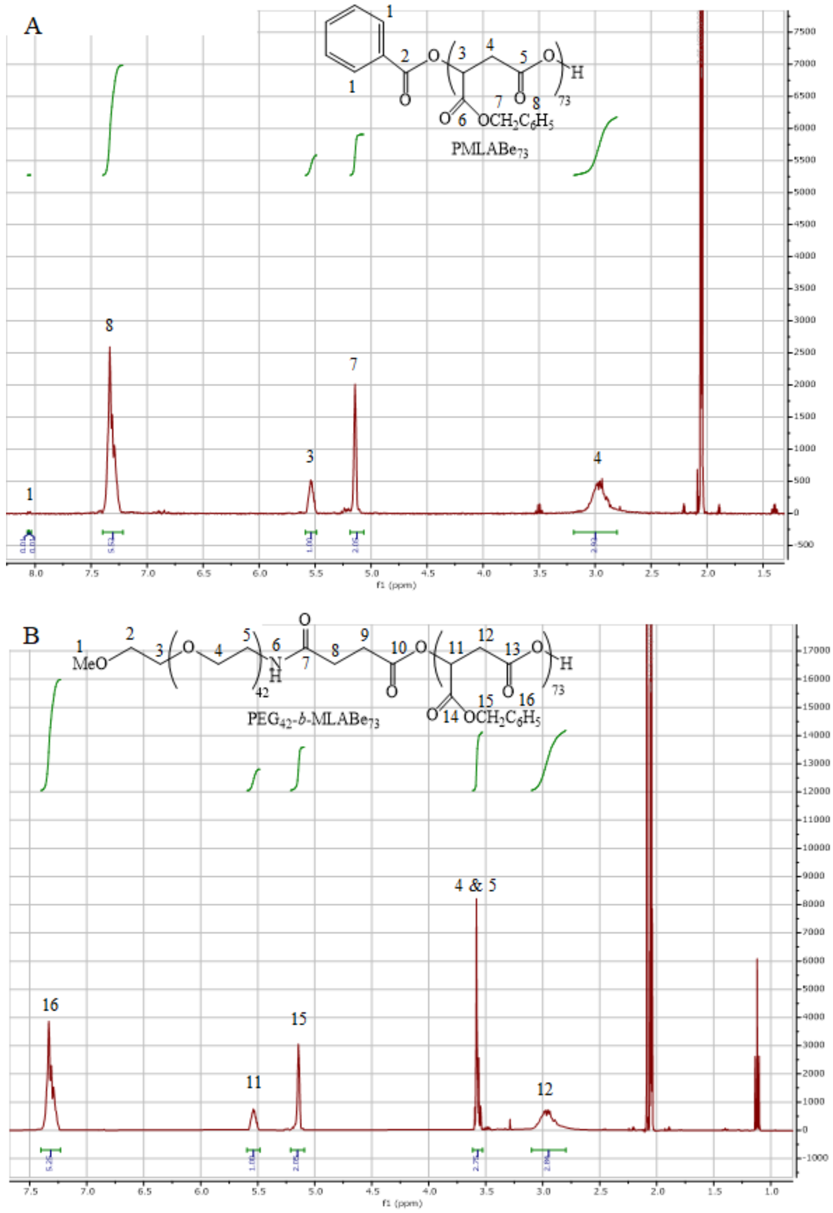

Figure S1. ^{1}H NMR spectra (CD_3COCD_3) of A. $PMLABe_{73}$, B. PEG_{42}-*b*-$PMLABe_{73}$.

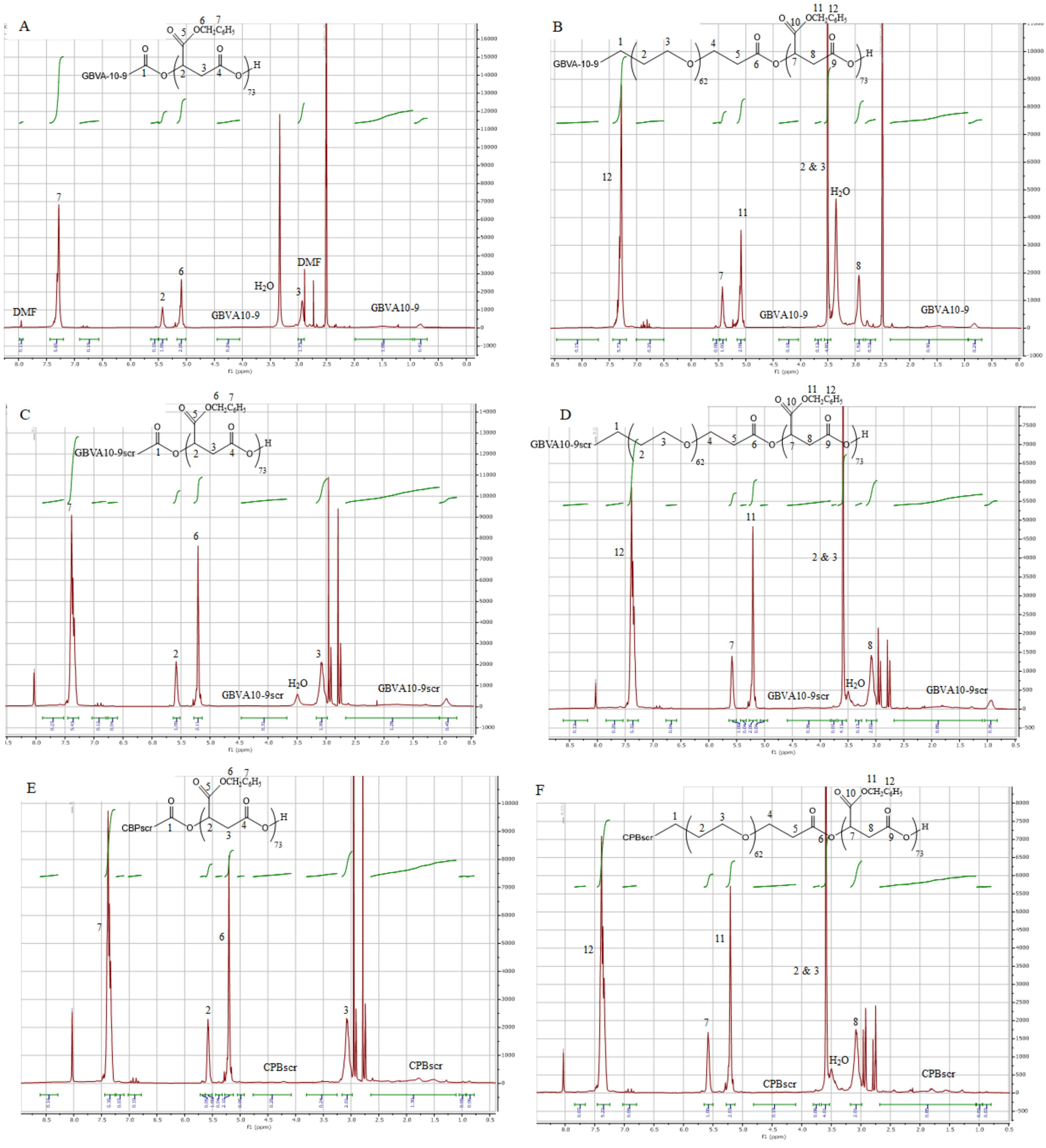

Figure S2. ^{1}H NMR spectra of A. GBVA10-9PMLABe$_{73}$ (DMSO-d6), B. GBVA10-9PEG$_{62}$-*b*-PMLABe$_{73}$ (DMSO-d6), C. GBVA10-9scrPMLABe$_{73}$ (DMF-d7), D. GBVA10-9scrPEG$_{62}$-*b*-PMLABe$_{73}$ (DMF-d7), E. CPBscrPMLABe$_{73}$ (DMF-7), E. CPBscrPEG$_{62}$-*b*-PMLABe$_{73}$ (DMF-d7).

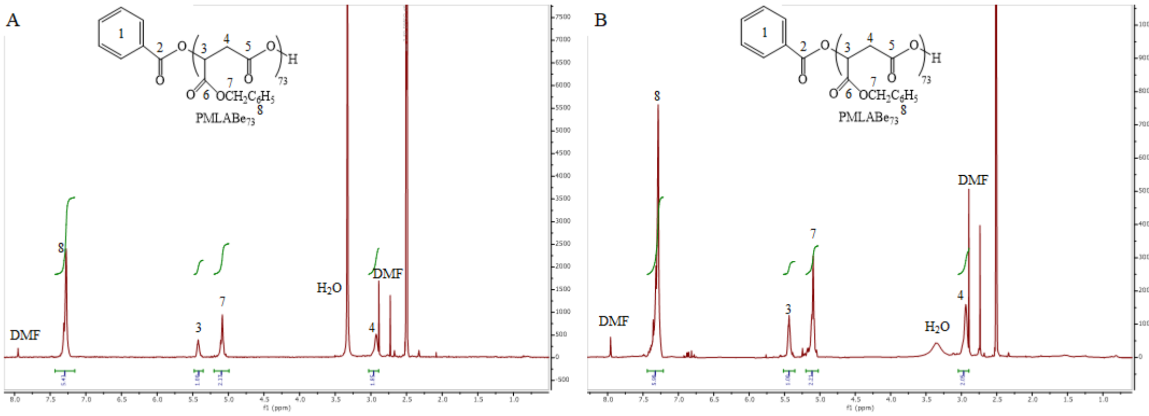

Figure S3. ^{1}H NMR spectra of products obtained after dialysis of: A. a mixture of PMLABe73 and GBVA10-9-SH, and B. a mixture of PMLABe73 and CPB-SH.
